# Arbuscular Mycorrhizal Fungi Negatively Affect Nitrogen Acquisition and Grain Yield of Maize in a N Deficient Soil

**DOI:** 10.3389/fmicb.2018.00418

**Published:** 2018-03-08

**Authors:** Xin-Xin Wang, Xiaojing Wang, Yu Sun, Yang Cheng, Shitong Liu, Xinping Chen, Gu Feng, Thomas W. Kuyper

**Affiliations:** ^1^Environment and Food Security, College of Resources and Environmental Sciences and Centre for Resources, China Agricultural University, Beijing, China; ^2^Land and Environmental College, Shenyang Agricultural University, Shenyang, China; ^3^Heilongjiang Academy of Agricultural Sciences Postdoctoral Program, Northeast Forestry/Agricultural University Postdoctoral Program, Harbin, China; ^4^Institute of Crop Tillage and Cultivation, Heilongjiang Academy of Agricultural Sciences, Harbin, China; ^5^Department of Soil Quality, Wageningen University and Research, Wageningen, Netherlands

**Keywords:** arbuscular mycorrhizal fungi, benomyl, maize, nitrogen uptake, competition

## Abstract

Arbuscular mycorrhizal fungi (AMF) play a crucial role in enhancing the acquisition of immobile nutrients, particularly phosphorus. However, because nitrogen (N) is more mobile in the soil solution and easier to access by plants roots, the role of AMF in enhancing N acquisition is regarded as less important for host plants. Because AMF have a substantial N demand, competition for N between AMF and plants particularly under low N condition is possible. Thus, it is necessary to know whether or not AMF affect N uptake of plants and thereby affect plant growth under field conditions. We conducted a 2-year field trial and pot experiments in a greenhouse by using benomyl to suppress colonization of maize roots by indigenous AMF at both low and high N application rates. Benomyl reduced mycorrhizal colonization of maize plants in all experiments. Benomyl-treated maize had a higher shoot N concentration and content and produced more grain under field conditions. Greenhouse pot experiments showed that benomyl also enhanced maize growth and N concentration and N content when the soil was not sterilized, but had no effect on maize biomass and N content when the soil was sterilized but a microbial wash added, providing evidence that increased plant performance is at least partly caused by direct effects of benomyl on AMF. We conclude that AMF can reduce N acquisition and thereby reduce grain yield of maize in N-limiting soils.

## Introduction

Soil microbes play crucial roles in nitrogen (N) transformations and turnover in soil. Microbial carbon (C) and N use efficiency in relation to N content of the substrate (both expressed through C:N ratios) drive these processes (Coleman et al., 2004). Demand for N by soil microorganism can generate competition between microbes and plants, with microbes being competitively superior (Hodge and Fitter, 2010). As a consequence of microbial competitive superiority, plant growth suppression has been reported for soils that contain low mineral N and to which a high C:N ratio substrate such as straw has been added. This mechanism has been frequently reported for saprotrophic microbes. This negative effect can be alleviated under conditions where arbuscular mycorrhizal fungi (AMF) successfully compete with saprotrophic microbes (Hodge et al., 2000; Nuccio et al., 2013). However, AMF also have a substantial N demand, as their biomass has a higher N concentration than that of plants (Helgason and Fitter, 2009; Hodge and Fitter, 2010). Competition for N between AMF and plants is therefore also possible; in cases of substantial N immobilization in AMF tissue, plant performance could be even reduced. In grassland ecosystems, competition for N between grass plants and AMF has been shown (Blanke et al., 2005; Püschel et al., 2016). It is unknown how often this competition for N between AMF and crops occurs in agro-ecosystems, or/and to what extent the competition may constrain plant production under conditions of N limitation.

AMF form symbiotic associations with more than two-thirds of the world's terrestrial plant species (Smith and Read, 2008). The enhanced acquisition of P by plants has generally been considered as the most important benefit of these fungi from an evolutionary perspective. The role of AMF in enhancing N acquisition is regarded as less important. Studies have reported that AMF are involved in N acquisition of plants. The extraradical mycelium of *Rhizophagus intraradices* expresses a functional high-affinity ammonium transporter (López-Pedrosa et al., 2006), implying that the extraradical mycelium can acquire ${\text{NH}}_{4}^{+}$ from soil. Fellbaum et al. (2012) demonstrated that the C supply by the host plant triggers the uptake and transport of N in the symbiosis. N exchange between fungus and plant in the symbiosis is stimulated only when the C is delivered by the host across the mycorrhizal interface, implying C–N trade between host plants and AMF. Feng et al. (2002) observed that AMF facilitate plant acquisition of N from sources that are not or hardly available to non-mycorrhizal plants. Hodge and Fitter (2010) claimed that AMF accelerated decomposition and acquired N from organic material. However, the balance between N immobilization by the mycorrhizal mycelium and N transfer to the plant as a function of soil N availability has been poorly understood, as is evident from recent reviews on the role of AMF in the N uptake of plants (Bücking and Kafle, 2015).

A limitation to field experiments that test effects of indigenous AMF on N acquisition is the near-impossibility of obtaining an appropriate “non-mycorrhizal control” for most plant species, because plants in nature are normally colonized. Recent studies demonstrated that even in intensive agriculture mycorrhizal colonization of crops can be high (Dinnes et al., 2002; Galván et al., 2009; Wang et al., 2015) and that AMF diversity can be substantial (Oehl et al., 2005; Hijri et al., 2006; Wang et al., 2015). Benomyl, [Methyl 1-(butylcarbamoyl)-2-(benzimidazole) carbamate; C_14_H_18_N_4_O_3_], which inhibits AMF enzyme activities and mycorrhizal colonization (Fitter and Nichols, 1988; Larsen et al., 1996), has been recommended as a treatment to reduce (but not fully eliminate) AMF colonization in the field (Fitter and Nichols, 1988).

In this study, we used benomyl to reduce mycorrhizal colonization of maize roots in order to test the effect of AMF on N uptake by maize plants in the field and in the greenhouse. In the greenhouse we further tested the effects of benomyl on plant N nutrition and growth in sterilized or non-sterilized soils, because benomyl can have several other direct and indirect effects on plant performance apart from its effects on AMF. We hypothesized that suppression of colonization by native AMF by benomyl would promote plant N uptake and thereby plant growth under low, but not under high N condition. The specific aim of this study was to understand the role of native AMF community in crop N uptake and biomass under field conditions with and without N fertilizer input.

## Materials and methods

### Field experiments

The trial site was located at the research station of China Agricultural University in Shangzhuang, Beijing, China. The annual average temperature in the region ranges from 11 to 13°C, and annual rainfall from 480 to 580 mm. The annual mean hours of sunshine are 2,700–2,800 h. There is a frost-free period of 180–200 days. Weather data during the maize-growing season are listed in Table S1. During the experiments in both years, sufficient water was supplied for plant growth besides rainfall.

Field experiments were conducted in 2007 and 2008 in two adjacent sites. The soil at the study site is a calcareous alluvial soil with a loamy silt texture. The chemical properties of the 0–30 cm soil layer of the study site in 2007 and 2008 were as follows: total N 0.74 and 0.83 g kg^−1^, extracted mineral N (N_min_) 7.7 and 8.3 mg kg^−1^, pH (in water) 8.0 and 8.1, Olsen-P 4.00 and 7.63 mg kg^−1^, NH_4_OAc extracted K 52.7 and 78 mg kg^−1^, organic carbon 8.4 and 8.4 g kg^−1^, soil bulk density 1.44 and 1.44 g cm^−3^, respectively. Maize hybrid, Denghai3719 (DH3719), was used as the test maize variety in both years. It was sown on April 29th, 2007 and May 9th, 2008, and harvested on September 14th, 2007 and September 28th, 2008, respectively. The field was irrigated before sowing in order to keep soil water content above 75%.

The experiment was set up as a randomized block design with two factors. The factors were N fertilizer-rate (0 or 450 kg N ha^−1^; –N or +N) and benomyl application (without or with benomyl, –B or +B). The high N-fertilizer treatment corresponds with the local conventional N fertilization. The experiment was carried out with four replicates, giving 16 plots in each year. Plot size was 3.92 (2.8 × 0.9 m) and 9.8 m^2^ (3.5 × 2.8 m) in 2007 and 2008, respectively.

Maize was over-seeded with hand planters and was thinned at the seedling stage to a stand density of 100,000 plants ha^−1^. Plant distance within rows was 28 cm, and distances between rows were 50 cm (wide row) alternating with 20 cm (narrow row). Border plots were included on the sides of the experimental field. Weed growth on plots was controlled during the experiment by manual labor.

Benomyl was produced by Taicang Chemical Co. Ltd., Jiangsu Province, China. This benomyl product contained 50% active benomyl ingredient and 6% N. Nine grams of benomyl (active ingredient) with 15 l water was supplied per m^2^ every 15 days until 105 DAS (days after sowing) in 2007 and every 20 DAS until 111 DAS in 2008, starting from 2 days before sowing. In all, benomyl was applied 7 and 6 times in 2007 and 2008, respectively. For non-benomyl treatments, 15 l water was given per m^2^ each time.

The rate and timing of N, P, and K fertilization were the same in both years. A total of 450 kg N ha^−1^ of urea was applied in +N treatments at different growth stages according to local farmers' practices. Urea was applied at a rate (kg N ha^−1^) of 175 at V0 stage (pre-sowing basal fertilization), 50 at V6 (6 leave stage), 170 at V10 (10 leaves stage), and 55 at VT (tasseling stage), respectively. Before sowing, 40 kg P ha^−1^ as triple superphosphate [Ca(H_2_PO_4_)_2_·H_2_O] and another 20 kg P was broadcasted at V12 (12 leaves stage). 66 kg K ha^−1^ as potassium sulfate (K_2_SO_4_) was broadcasted and incorporated into the upper 0–15 cm of the soil by rotary tillage. Another 33 kg ha^−1^ of K at VT was applied. Also Zn (10 kg ha^−1^) as ZnSO_4_·7H_2_O was applied in each year as base fertilizer because of the Zn deficiency in the region on these calcareous soils. For –N treatment, no N fertilizer was applied, while P and K fertilizer were applied as in +N treatment.

### Plant and soil sampling

Plants were harvested 57 (V10) and 147 (R6; physiological maturity, when 50% of the plants showed black-layer formation in the grains from the mid-portion of the ears) DAS in 2007. In 2008 plants were harvested 50 (V6), 68 (V12), 88 (silking, VT-R1), 111 (milking, R3), and 142 (R6) DAS. At every harvest, six neighboring plants (three plants each within the binate rows) were cut at the stem base, chopped to a fine consistency, dried to a constant weight at 60°C and ground. Shoot samples were digested in a mixture of concentrated H_2_SO_4_ and H_2_O_2_. Digests were analyzed for N by the Kjeldahl method and for P by the molybdo-vanadophosphate method (Johnson and Ulrich, 1959; Shi, 1986). Shoot N concentration was only assessed in 2008.

After cutting off shoots at each harvest, a soil volume of 28 × 35 × 30 cm (depth) was dug out. There were three soil samples for each plot. All visible roots in each soil sample were collected by hand and placed in individual labeled plastic bags. Roots were washed free of soil after transfer to the laboratory and then frozen at −20°C. Frozen roots were cut into 1-cm segments and thoroughly mixed. A 0.5-g subsample was cleared with 10 % (w/v) KOH at 90°C for 2 h and stained with trypan blue for quantification of mycorrhizal colonization (Trouvelot et al., 1986).

### Pot experiments

#### Pot experiment 1

In order to test for a potential nutritional effect by N contained in benomyl on plant growth, we conducted greenhouse pot experiment 1 in 2008. The experiment was a randomized complete full factorial block design with two factors: (1) soil sterilization—two levels, non-sterilized (NS) and steam-sterilized with subsequent addition of a microbial wash (S); (2) benomyl—two levels, without (–B) and with benomyl (+B). There were four treatments, with each treatment having four replicates, giving 16 pots. We harvested maize plants 58 DAS.

The fungicide pots received benomyl as a soil drench, whereas the control pots received water. The benomyl was purchased from the same company. For treatments of NS+B and S+B, an emulsion of 1.2 g active ingredient of benomyl in 2 l water was uniformly applied 2 days before sowing, while 2 l of water was applied for treatments of NS-B and S-B. The pots were drenched every 2 weeks. The soil was collected from the same site as the field experiment in 2008. The soil was passed through a 2-mm sieve and sterilized by steam-sterilization for 2 h at 121°C. The soil was then placed in porcelain pots, 20 cm height, 30 cm diameter, with 4 kg of soil pot^−1^. There was a hole at the bottom of the pot. The leachate from each pot was collected and reintroduced to the pot. To minimize differences in the microbial communities of sterilized and non-sterilized treatments, 20 ml of AMF-free filtrate, filtered through a 10 μm membrane filter, taken from 20 g native soil and 200 ml deionized water was added to each sterilized pot, and 20 ml of deionized water was added to each non-sterilized pot. The basal fertilizers were: 20 mg P (as KH_2_PO_4_), 200 mg N (as KNO_3_), 50 mg Mg (as MgSO_4_), 5 mg Zn (as ZnSO_4_), and 2 mg Cu (as CuSO_4_) kg^−1^ soil. The nutrients were mixed with the soil before potting. Three weeks after sowing, additional100 mg N (as KNO_3_) kg^−1^ soil was supplied with water to each pot.

Maize seeds (Denghai3719) were surface-sterilized in a 10% (v/v) solution of H_2_O_2_ for 10 min and then thoroughly washed with deionized water. Three seeds were sown in each pot. Seedlings were thinned to one when the third leaf appeared. The seedlings were irrigated with deionized water and soil moisture was maintained at 60–70% of water holding capacity after weighing twice per week. The plants grew with a daytime temperature range of 25–32°C and a night temperature range of 20–25°C. The pots were arranged randomly within a block in the glasshouse, with the positions re-randomized every week.

#### Pot experiment 2

In order to repeat the effects of benomyl on AMF and subsequent effects on N and P uptake and maize growth, we conducted a second greenhouse pot experiment. The experiment was set up in a randomized complete full factorial block design. There were two treatments with non-sterilized soil, i.e., without (–B) and with benomyl (+B). The experiment was carried out with eight replicates, giving 16 pots in total. We harvested twice at the 27th and 58th day after sowing. Every time, we harvested four replicates per treatment.

The procedure and basic information of Pot experiment 2 was the same as Pot experiment 1, except that benomyl was applied every 3 weeks.

### Harvest and measurements of the pot experiments

Plants were harvested at 58 DAS in Pot 1, and at 27 and 58 DAS in Pot 2. Plants were separated into shoots and roots. The shoots were oven-dried at 72°C for 48 h and finally ground. Roots were washed with deionized water and then preserved at −20°C. Mycorrhizal colonization was measured as in the field. Plant N and P concentration was determined by the same method used in the field experiment.

### Statistics

Data are presented as arithmetic mean values with standard errors. Data met requirements of homogeneity of variance (Levene's test) except grain yield in 2008. After log-transformation these data also met the ANOVA assumptions. Two-way ANOVA was used to analyze the interaction between N fertilizer and benomyl in the field experiment, and between sterilization and benomyl in pot experiment 1. Two-way ANOVA was used for the field experiments at each sampling time and pot experiment 1, whereas on-way ANOVA was used for pot experiment 2. Differences at the 5% level of significance were compared through Tukey's Honestly Significant Difference (HSD). Correlation between variables was tested using Pearson's correlation coefficient (*P* < 0.05). Statistical analyses were performed with SPSS software, version 20.0 (IBM Corp. in Armonk, NY., USA).

## Results

### Effect of benomyl on maize performance

**Figure 1:**
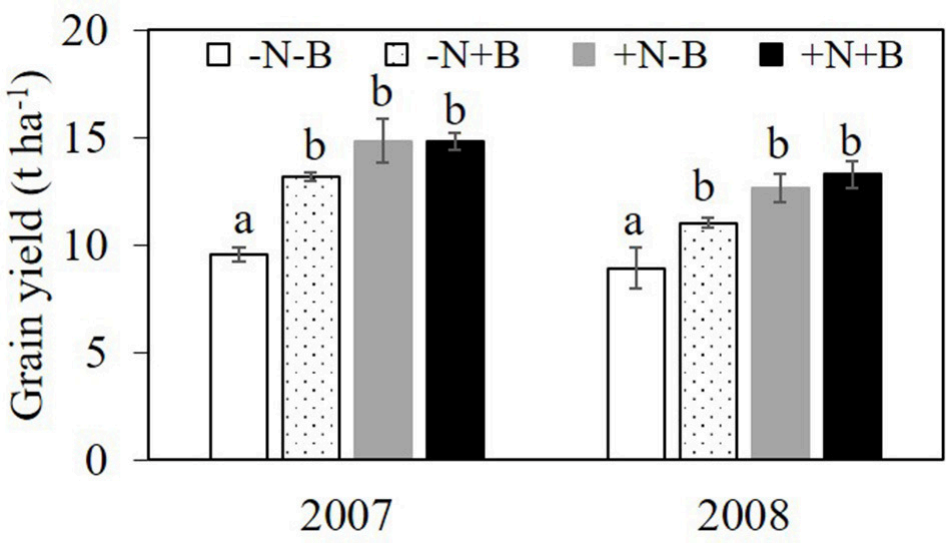
Grain yield (t ha^−1^) in the field experiment in 2007 and 2008. Effects of N fertilizer supplies and benomyl on maize grain yield. Each value is the mean of four replicates (±SE). Different letters above bars denote significant difference among treatments at a specific growth stage (*P* < 0.05). –N–B: no N fertilizer and no benomyl; –N+B: no N fertilizer and benomyl; +N–B, N fertilizer and no benomyl; +N+B, N fertilizer and benomyl.

The effects of N and benomyl on plant biomass in 2007 and 2008 were somewhat inconsistent. Nitrogen was usually a significant source of variation, whereas benomyl was a significant source in 2007 at both sampling dates and in 2008 only after the first sampling. The interaction between N and benomyl was significant in the first sampling (2007) and the final sampling (2008; Tables 1, 2). In the field without N fertilizer input, maize plants manifested slight N deficiency symptoms by visual observation. N fertilizer generally increased shoot biomass (Figure S1). Grain yield was affected by N in both years (*P* < 0.001, in 2007; *P* < 0.01, in 2008), and by benomyl, especially in 2007 (*P* < 0.01 and *P* < 0.05, in 2007 and 2008, respectively). Application of benomyl increased grain yield in the absence of N with 38 and 23%, respectively, and had no effect when N fertilizer was applied (Tables 1, 2; Figure 1).

**Table 1:** ANOVA results with nitrogen fertilizer input (N), and benomyl (B) as independent variables, and shoot biomass, P concentration, P content, colonization, and grain yield as dependent variable on different sampling days in 2007.

**After sowing**	**Source**	***df***	**Biomass**	**P concentration**	**P content**	**Colonization**	**Grain yield**
58 DAS	Nitrogen (N)	1	23.682^***^	0.094	13.596^**^	0.931	
	Benomyl (B)	1	5.338^*^	1.324	11.838^**^	28.514^*^	
	N ^*^ B	1	8.469^*^	1.473	3.254	0.028	
147 DAS	Nitrogen (N)	1	3.146	0.701	0.812	6.591^*^	36.878^***^
	Benomyl (B)	1	5.719^*^	1.325	6.763^*^	20.955^**^	9.939^**^
	N ^*^ B	1	0.156	0.019	0.064	22.935^***^	10.421^**^

*F-values are shown*.

*, **, *** *, presents significance at P < 0.05, P < 0.01, P < 0.001, respectively*.

**Table 2:** ANOVA results with nitrogen fertilizer input (N), and benomyl (B) as independent variables, and shoot biomass, N and P concentration, N and P content, colonization and grain yield as dependent variable on different sampling days in 2008.

**After sowing**	**Source**	***df***	**Biomass**	**P concentration**	**N concentration**	**N:P**	**P content**	**N content**	**Colonization**	**Grain yield**
68 DAS	Nitrogen (N)	1	10.39^**^	31.787^***^	105.875^***^	5.443^*^	59.923^***^	124.068^***^	0.021	
	Benomyl (B)	1	22.026^**^	9.929^**^	18.492^**^	29.077^***^	41.704^***^	0.147	65.287^***^	
	N ^*^ B	1	0.512	1.31	3.037	0.251	0.985	1.524	0.025	
88 DAS	Nitrogen (N)	1	11.223^**^	22.517^***^	180.581^***^	3.514	30.896^***^	85.149^***^	22.81^***^	
	Benomyl (B)	1	0.002	0.166	21.186^**^	5.100^*^	0.227	5.13^*^	18.667^**^	
	N ^*^ B	1	0.656	0.368	1.136	0.311	0.978	0.025	0.845	
111 DAS	Nitrogen (N)	1	5.829^*^	37.729^***^	102.894^***^	24.743^***^	38.393^***^	58.099^***^	16.34^**^	
	Benomyl (B)	1	0.263	0.156	7.726^*^	10.739^**^	0.707	0.915	118.781^***^	
	N ^*^ B	1	1.605	7.16^*^	14.394^**^	31.224^***^	0.168	9.529^**^	4.781^*^	
141 DAS	Nitrogen (N)	1	4.382	0.256	123.718^***^	82.041^***^	0.134	129.571^***^		18.951^**^
	Benomyl (B)	1	0.341	2.988	0.561	3.713	1.935	0.735		4.435^*^
	N ^*^ B	1	6.334^*^	2.178	8.162^*^	1.51	4.093	16.727^**^		1.211

*F-values are shown*.

*, **, *** *, presents significance at P < 0.05, P < 0.01, P < 0.001, respectively*.

**Figure 2:**
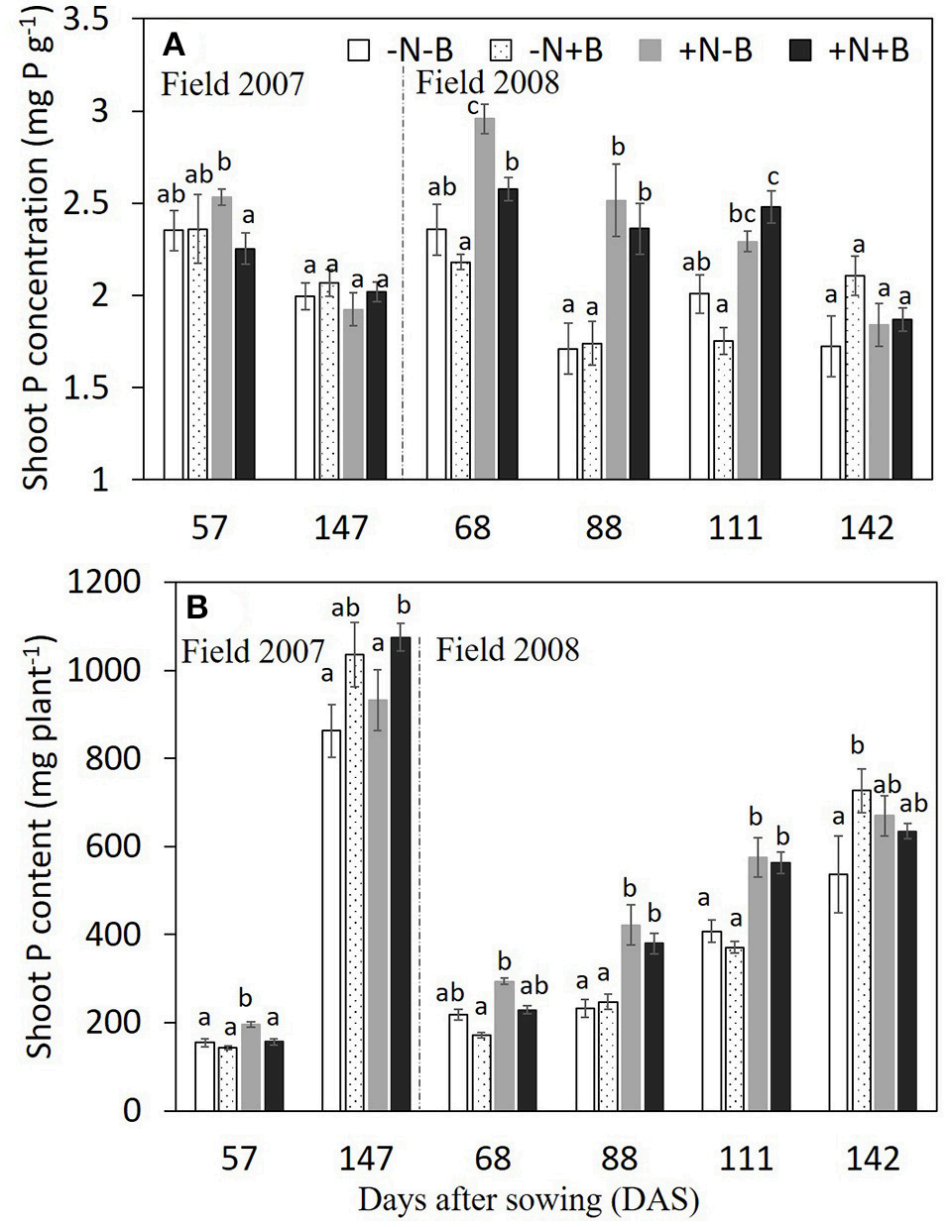
Effects of N fertilizer supplies and benomyl on shoot P concentration **(A)** and shoot P content **(B)** by maize at different growth stages in 2007 (and 2008). Each value is the mean of four replicates (±SE). Different letters denote significant difference (*P* < 0.05) among the treatments at each growth stage. –N–B: no N fertilizer and no benomyl; –N+B: no N fertilizer and benomyl; +N–B, N fertilizer and no benomyl; +N+B, N fertilizer and benomyl. DAS: days after sowing for years 2007 (2008) [57 (68) = jointing; 88 = silking; 111 = milk: 147 (142) = harvest].

**Figure 3:**
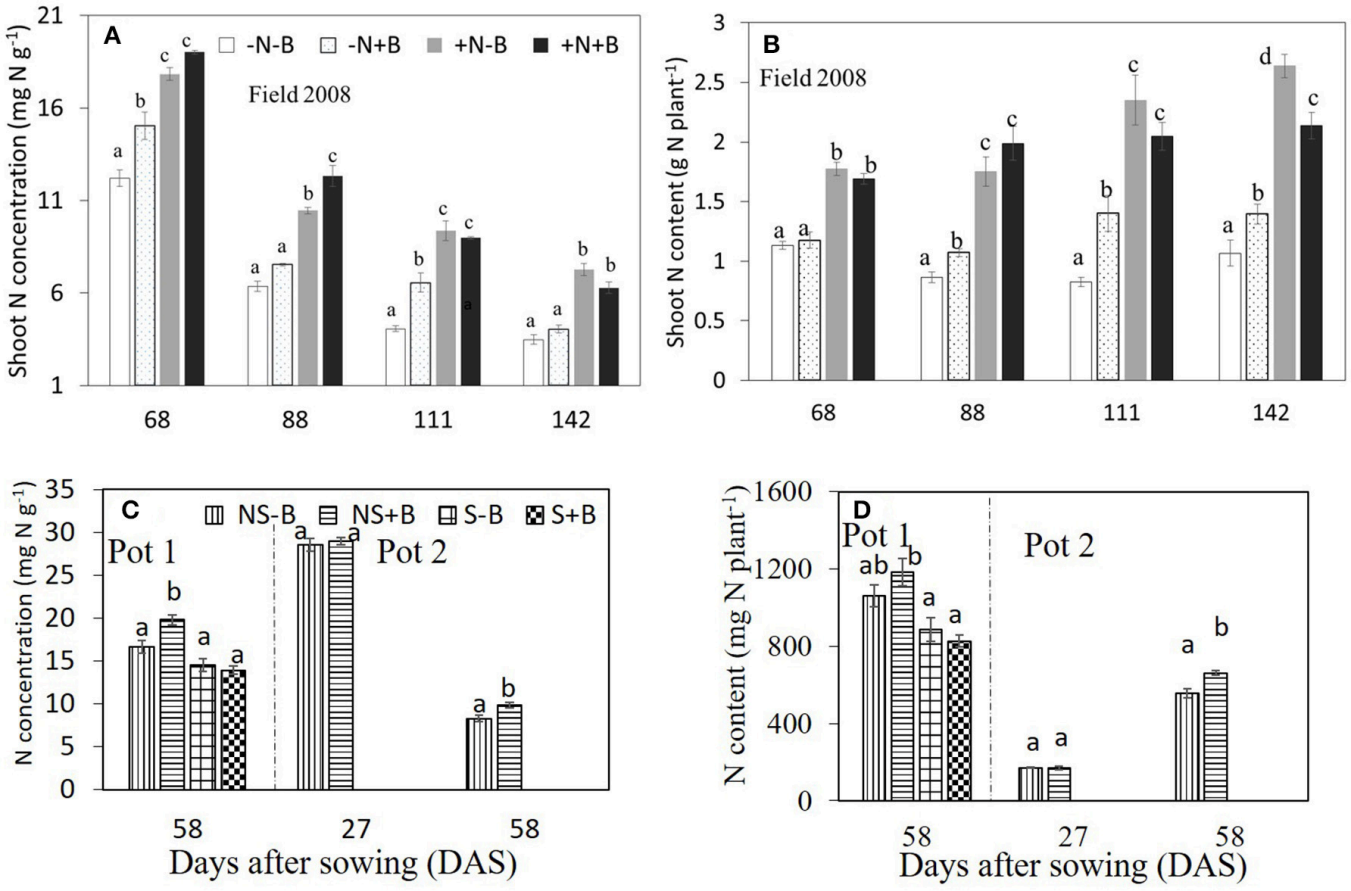
Effects of N fertilizer supplies and benomyl on N concentration **(A)** and **(C)**, and N uptake **(B)** and **(D)** by maize at different growth stages in the field (**A,B**; in 2008) and in the pots **(C,D)** experiments. Each value is the mean of four replicates (±SE). –N–B: no N fertilizer and no benomyl; –N+B: no N fertilizer and benomyl; +N–B, N fertilizer and no benomyl; +N+B, N fertilizer and benomyl. NS–B: non-sterilized soil and no benomyl; NS+B: non-sterilized soil and benomyl; S–B, sterilized soil and no benomyl; S+B, sterilized soil and benomyl. Different letters denote significant difference (*P* < 0.05) among the treatments at each growth stage.

Neither N nor benomyl nor the interaction was a significant source of variation of P concentration in 2007 (Table 1; Figure 2). In 2008 N was a significant source of variation, except for the final harvest, whereas benomyl was only a significant source of variation 68 DAS and the interaction N and benomyl was only significant 111 DAS (Table 2; Figure 2). Plant P content showed the same pattern as reported for biomass in 2007 and 2008 (Figure 2). Shoot N concentration was only assessed in 2008. It was significantly affected by N fertilization at all harvests, and by benomyl in three (of four) sampling times (no effect at 141 DAS), whereas the interaction between N and benomyl was significant at 111 and 141 DAS (Table 2; Figure 3). Shoot N content was significantly affected by N fertilization, whereas benomyl was usually not a significant source of variation. The N × benomyl interaction was significant at both 111 and 141 DAS (Figures 3A,B).

Shoot N:P ratios were significantly affected by benomyl treatments (only at 141 DAS was the difference marginally not significant; Table 2) and N-fertilization, especially at the later stages. Application of benomyl increased N:P ratios.

Soil sterilization (elimination of AMF) in pot experiment 1 had a significant effect on shoot P and N concentration, shoot P and N content, and shoot N:P ratio, but not on shoot biomass (Table 3). Benomyl was not a significant source of variation. There was a significant interaction between sterilization and benomyl for both shoot N and P concentration (Table 3; Figure S2). Soil sterilization reduced shoot P concentration, but did not influence maize shoot biomass. Sterilization also reduced shoot N concentration, and this effect was largest in the benomyl treatment (significant interaction between sterilization and benomyl; Figure 3). The negative effect of soil sterilization on shoot N and P concentration implies that AMF did have an effect on nutrient uptake. Shoot N content followed the same pattern as N concentration (Figure 3). In sterilized soil, shoot N:P ratios ranged between 12 and 13.

**Table 3:** ANOVA results with nitrogen soil sterilization, and benomyl (B) as independent variables, and shoot biomass, N and P concentration, N and P content as dependent variable in Pot experiment 1.

**Source**	***df***	**Biomass**	**P concentration**	**N concentration**	**N:P**	**P content**	**N content**
Sterilization (S)	1	0.456	409.401^***^	36.282^***^	101.896^***^	156.217^***^	21.886^**^
Benomyl (B)	1	1.166	1.392	3.872	0.805	0.015	0.322
S ^*^ B	1	0.224	5.737^*^	8.078^*^	0.011	0.746	2.537

*F-values are shown*.

*, **, *** *, presents significance at P < 0.05, P < 0.01, P < 0.001, respectively*.

### Mycorrhizal colonization

**Figure 4:**
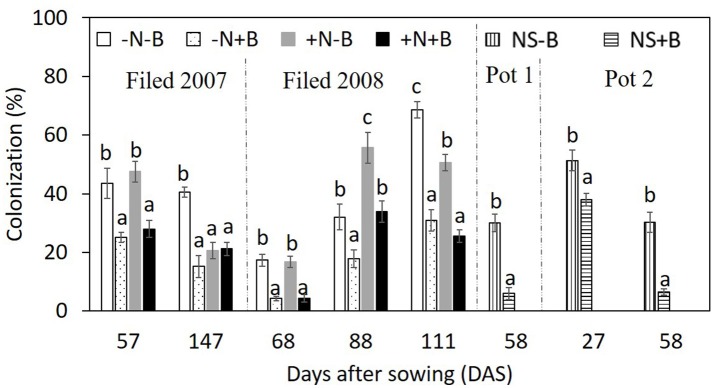
Effects of N fertilizer supply and benomyl on colonization by maize at different growth stages in the field experiment in 2007 and 2008, and in the pot experiment 1 and 2. Each value is the mean of four replicates (±SE). Different letters denote significant difference (*P* < 0.05) among the treatments at each growth stage. –N–B: no N fertilizer and no benomyl; –N+B: no N fertilizer and benomyl; +N–B, N fertilizer and no benomyl; +N+B, N fertilizer and benomyl. DAS: days after sowing for years 2007 (2008) [57 (68) = jointing; 88 = silking; 111 = milk: 147 = harvest]; NS–B: non-sterilized soil and no-benomyl; NS+B: non-sterilized soil and benomyl in Pot 1 and 2.

**Figure 5:**
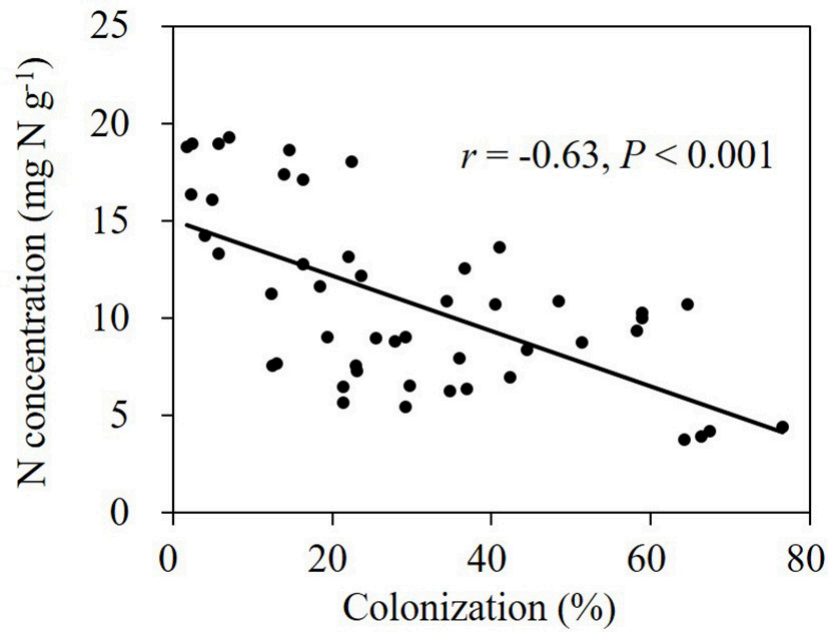
Relationship between shoot N concentration and mycorrhizal colonization by native AMF community in the field experiment in 2008 (Person, *P* < 0.05).

Application of benomyl significantly reduced mycorrhizal colonization in the field (Tables 1, 2; Figure 4). The effect of N fertilizer on colonization was variable between years and growth stages: it reduced colonization at 147 DAS in 2007, increased colonization at 88 DAS in 2008, but reduced colonization at 111 DAS in the same year. There was also a significant interaction between N and benomyl treatment in several plant growth stages a for example, at 147 DAS in 2007 and at 111 DAS in 2008 (Tables 1, 2). It was also found that benomyl greatly suppressed arbuscular formation (Figure S3). There was a positive correlation between arbuscular (%) and colonization (%) (*y* = 0.64*x* + 1.5; *r* = 0.74; *P* < 0.001) using the field data 2008. Mycorrhizal colonization in the field by native AMF and shoot N concentration were significantly negatively correlated in 2008 (*r* = −0.63, *P* < 0.001; Figure 5). There was also a significant negative correlation between mycorrhizal colonization and shoot N:P ratio in 2008 (*r* = −0.71; *P* < 0.001; Figure S4). We did not observe visible signs of pathogen damage in the treatments.

In pot experiment 1, we did not observe any colonization in sterilized soil regardless of application of benomyl, indicating that AMF were effectively eliminated. Benomyl again reduced mycorrhizal colonization from 30 ± 3 to 6 ± 2% (Figure 4) at 58 DAS after sowing. In pot experiment 2, this negative effect was stronger after 58 DAS than after 27 DAS (Figure 4). At 27 DAS, benomyl reduced root colonization from 51 ± 4 to 38 ± 2%; at 58 DAS, benomyl reduced root colonization from 30 ± 4 to 6 ± 1% (Figure 4).

## Discussion

Our field experiment showed, for both 2007 and 2008, that the application of benomyl increased maize yield and N uptake under conditions of low N availability but not under conditions of high N availability. Mycorrhizal colonization was suppressed by benomyl, irrespective of the fertilizer treatment. These result therefore confirmed the hypothesis of our study. The pot experiments provided further support for the causal nexus between alleviation of N immobilization in AMF mycelium through the application of benomyl and the enhanced plant performance under conditions of N limitation.

A crucial prerequisite for the test of our hypothesis is that the effect of AMF decreasing maize N content is solely due to inhibition of mycorrhizal colonization by benomyl. However, benomyl may affect plant growth and N acquisition via a number of possible mechanisms.

First, benomyl suppresses the activity of AMF (Trappe et al., 1984; Fitter and Nichols, 1988). In the presence of AMF (both in the field and greenhouse), benomyl did significantly reduce mycorrhizal colonization. Benomyl also increased plant N:P ratios in the field, indicating either reduced P-uptake due to reduced mycorrhiza activity and/or enhanced N-uptake due to reduced N immobilization in the mycorrhizal fungal mycelium. Increases in biomass and N uptake by maize at low, but not at high N availability suggest that mycorrhizal fungal suppression by benomyl is the causal factor for the observed effects. Several studies did report a limited effect of benomyl application in the field. Pedersen and Sylvia (1997) reported that a field applications of 5–20 kg ha^−1^ did not reduced colonization of a mycorrhizal grass. However, the amounts used where much smaller than the dose applied by us; furthermore these authors and applied benomyl as a foliar spray. They suggested that a soil drench would be preferable over a foliar spray, and a soil drench was used by us. Larsen et al. (1996) also determined that a foliar spray of benomyl had very different effects than the use of a soil drench, with negative effects of benomyl on the P uptake only occurring after soil application.

Second, benomyl may promote plant growth through inhibition of soil pathogens (Newsham et al., 1994; Smith et al., 2000; Hodge and Fitter, 2013) and so a reduced pathogen pressure could also have caused the growth stimulation of benomyl. However, we did not observe pathogen damage in the field, which is likely due to the extensive pathogen control in the experimental station. We also did not observe pathogen damage in the pot experiment 1. AMF have been reported to suppress fungal pathogens and a decline in AMF activity due to benomyl could as well have had positive effects on pathogens. For that reason we consider it unlikely that the second mechanism constitutes the main explanation. However, further studies into the way into which benomyl directly affects AMF and directly and indirectly (through AMF) affects soil pathogenic fungi may merit future studies.

Third, benomyl may affect plant N nutrition directly through microbial degradation of benomyl and N mineralization. Benomyl contains N (6%) and its half-life ranges from <2 up to 7 weeks (Lee et al., 2004). Observations by O'connor, Manjarrez and Smith (2009) that repeated applications of benomyl did not have cumulative negative effects on AMF were explained by these authors by the rapid degradation of the fungicide as well. In our study, maximum N addition due to benomyl application rate was around 38 and 32 kg N ha^−1^ in 2007 and 2008, respectively, which is < 10% of the difference in N fertilizer addition between –N (0 kg N ha^−1^) and +N (450 kg N ha^−1^) if all benomyl was degraded, or even far <10% if only part of the benomyl was degraded. Benomyl may also have impacted on saprotrophic fungi reducing, at this high application rate, decomposition and soil respiration, thereby decreasing net N mineralization (Chen et al., 2001a,b). Torstensson and Wessen (1984) reported negative effects of benomyl on several pathogenic (*Verticillium, Fusarium*) and saprotrophic fungi. Our results show that benomyl application significantly enhanced shoot N concentration, shoot N content and shoot biomass in the non-sterilized soil, but had no effects in sterilized soil (Figures 3C,D; Figure S1B). We therefore exclude the possibility that the benomyl effects were due to its function as a direct N source.

Fourth, benomyl may stimulate plant growth, having cytokinin-like effects, reducing leaf senescence (Garcia et al., 2003), and improving plant biomass in some species (Allison et al., 2007). Schweiger et al. (2001) also reported non-significant positive effects of benomyl on plant growth and no effect on root colonization at recommended dose (>0.5 l ha^−1^), and similar non-significant effect of benomyl on plant biomass while root colonization substantially declined. They suggested that simultaneous effects on other, more sensitive components of the soil microbial community with which AMF interact could have caused this effect. Our study shows that in sterilized soil, shoot N concentration did not differ between treatments with or without benomyl application (Figure 3C), indicating that benomyl application may not have stimulated the growth and N uptake of maize.

We therefore conclude that a beneficial effect of benomyl at low N availability, but no effect of benomyl at high N availability, is best explained by a negative effect of benomyl on AMF resulting in reduced N immobilization in AMF mycelium and hence reduced competition for N between AMF and plants, as proposed in our hypothesis. Further support for our hypothesis can be found in the observation that the benomyl effect disappeared after AMF were eliminated by soil sterilization, but where the microbial wash could have added other microbiota, including pathogenic fungi.

Colonization of maize roots was negatively correlated with shoot N concentration at both N levels (*r* = −0.63, *P* < 0.001; Figure 5). Colonization of maize roots was equally negatively correlated with N:P ratios in maize shoots when the data from two N levels were pooled regardless benomyl was applied or not (*r* = −0.71, *P* < 0.001; Figure S3). In the presence of AMF, shoot N:P ratios ranged between 6 and 10, which is indicative for N-limitation (Koerselman and Meuleman, 1996; Güsewell, 2004). In non-mycorrhizal soil N:P ratios ranged between 12 and 13, consistent with alleviation of N-limitation. Next to negative effects of benomyl on root colonization, Sukarno et al. (1993) and Kling and Jakobsen (1997) reported that benomyl also inhibited the growth of the external mycorrhizal mycelium.

An earlier study of *Artemisia vulgaris* equally showed negative correlations between mycorrhizal colonization and tissue N concentration and N:P ratio (Blanke et al., 2005). Reynolds et al. (2005) suggested that some AMF species can depress growth of plant species at low N supply. Hodge and Fitter (2010) found that AMF can obtain substantial amounts of N; this N is immobilized in the fungal tissue. N concentration of mycelium is on average 5–10 times as high as that of plant shoots and roots. Püschel et al. (2016) claimed that plant–fungus competition for N erased mycorrhizal growth benefits of *Andropogon gerardii* under limited N supply. These data contribute to a growing body of knowledge that alleviation of competition for nitrogen between AMF and plants can result in enhanced N uptake and plant performance.

Benomyl does not have a direct effect on soil P availability and therefore has no direct effect on maize growth in the non-mycorrhizal condition (Fitter and Nichols, 1988; Carey et al., 1992). Thus, the lower P concentration in shoots in the treatment of +N+B in the field experiment both in 2007 and in 2008 at earlier stage (Figure 2) is consistent with a mechanism whereby benomyl reduces mycorrhizal functioning (as evidenced by lower mycorrhizal colonization). Biweekly (triweekly) application of benomyl successfully reduced root colonization, which has been repeatedly shown to correlate with shoot P uptake (Lekberg and Koide, 2005; Chu et al., 2013). A complete inhibition of P uptake by hyphae occurred even within 5 DAS after benomyl was applied (Larsen et al., 1996). However, after the early harvest in both 2 years, shoot P concentration in the −B treatments was not significantly higher than that in the +B treatments regardless of N application. Apparently, the negative effect of benomyl on mycorrhiza-mediated plant P uptake can be transient. On the other hand, in pot experiment 1, benomyl had no significant effects on both P concentration and shoot biomass (Table 3; Figures S2B, S3). The soil used in pot experiment 1 contained 7.63 mg Olsen-P kg^−1^, and another 20 mg KH_2_PO_4_-P was added. Possibly higher available soil P content reduced mycorrhizal responsiveness. Wilson and Williamson (2008), when using Topsin-M, a fungicide with a similar mode of action as benomyl, reported that the fungicide significantly reduced mycorrhizal colonization in two grass species at two levels of soil fertility (with and without NP fertilizer addition), but the effect of the fungicide on plant performance was only significant for one plant species in the unfertilized treatment.

Our study was primarily designed to test the hypothesis of competition for mineral N between AMF and plants, whereby under conditions of N limitation alleviation of that competition would result in enhanced maize performance. In order to test that hypothesis we used high amounts of benomyl, more than 100 times the recommended dose. It is therefore difficult to directly translate the results of this study into practical recommendations for N management in agricultural systems in China. Current fertilizer recommendations for northern China (Peng et al., 2013) for maximum grain yield at optimal N fertilizer input are currently based on a critical soil N_min_-value. These recommendations do not consider microbial N demand from saprotrophic and mycorrhizal fungi. Our results indicate that under conditions of N limitation, as in various forms of low-input agriculture, AMF-mediated N-limitation can occur. Apparently, both ectomycorrhizal forests (Franklin et al., 2014; Kuyper and Kiers, 2014) and arbuscular mycorrhizal agro-ecosystems can be subject to a mycorrhizal trap.

## Author contributions

X-XW: analyzed the data, wrote the first manuscript, and modified it; XW, YS, YC, and SL: performed the experiments, collected the samples, and collected the data; XC: assisted to design the field experiments and managed the field experiments' control; GF: designed the experiments, revised the manuscript, and applied funding to support the study; TK: wrote and revised the manuscript, and modified the language. All the authors discussed the results and commented on the manuscript.

### Conflict of interest statement

The authors declare that the research was conducted in the absence of any commercial or financial relationships that could be construed as a potential conflict of interest.
