# Supplementary material for: Arbuscular Mycorrhizal Fungi Negatively Affect Nitrogen Acquisition and Grain Yield of Maize in a N Deficient Soil
Source: Front Microbiol. 2018 Mar 8;9:418. doi: 10.3389/fmicb.2018.00418 (PMC5852317; doi:10.3389/fmicb.2018.00418)
Supplement: Supplementary file 1 [file Presentation1.PDF]

Arbuscular mycorrhizal fungi negatively affect nitrogen acquisition and grain yield of maize in a N deficient soil

Xin-Xin Wang[1, 2 §], Xiaojing Wang[1 §], Yu Sun[3, 4 §], Yang Cheng[1], Shitong Liu[1],
Xinping Chen[1], Gu Feng[1*] and Thomas W. Kuyper[5]

Supporting materials

Table S1 | The weather data during the maize growing season at the research station of China Agricultural University, Shangzhuang, North China, near the Pacific Coast (40 °N, 116 °E).

Month	Temperature (°C)		Rainfall (mm)		Solar radiation (MJ m^{-2} d^{-1})	
	2007	2008	2007	2008	2007	2008
May	12.1-27.7	14.8-26.3	40.4	28.7	29.8	9.4
June	18.3-30.7	19.0-27.8	75.1	70.7	15.8	6.6
July	20.7-31.4	22.9-31.8	182.8	175.6	13.6	7.4
August	18.5-31.3	22.3-30.9	59.3	182.2	15	9.4
September	13.0-26.4	16.9-26.4	57	48.7	13.2	13

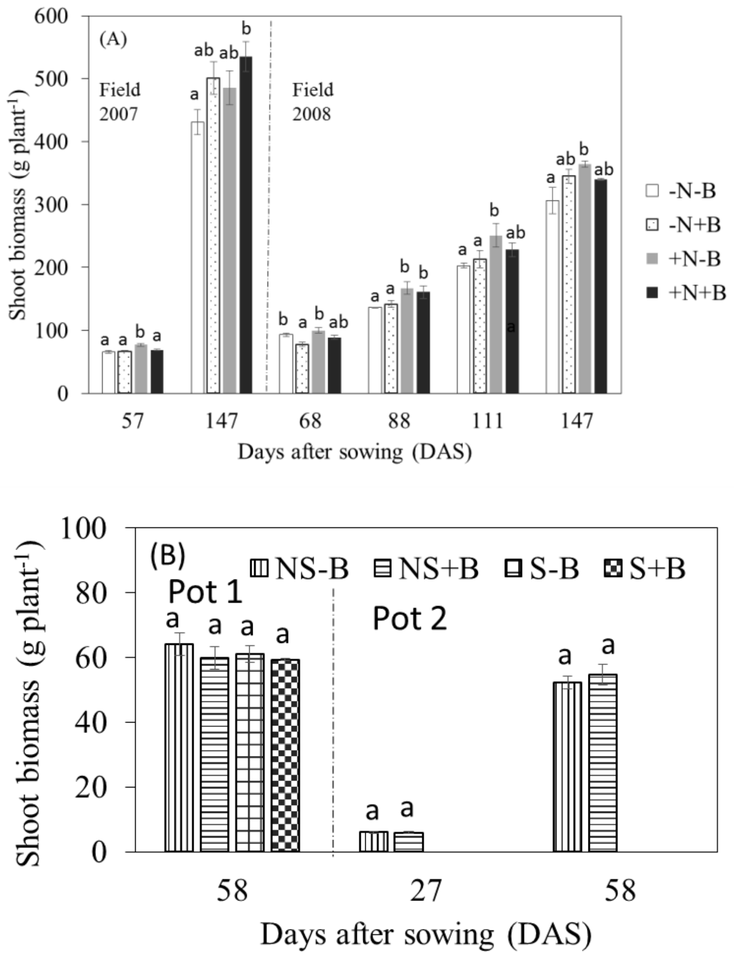

Figure S1 | Shoot dry weight of maize (g plant^{-1}) in different growth stages in 2007 and 2008 (A). Each value is the mean of four replicates (± SE). Different letters above bars denote significant difference among treatments at a specific growth stage ($P < 0.05$). -N-B: no N fertilizer and no benomyl; -N+B: no N fertilizer and benomyl; +N-B, N fertilizer and no benomyl; +N+B, N fertilizer and benomyl. DAS: days after sowing for years 2007 (2008) [57 (68) = jointing; (88) = silking; (111) = milking; 147 (142) = harvest]. Shoot dry weight of maize (g plant^{-1}) in Pot 1 and 2 (B). Each value is the mean of four replicates (± SE). NS-B: non-sterilized soil and without benomyl; NS+B: non-sterilized soil and with benomyl; S-B, sterilized soil and without benomyl; S+B, non-sterilized soil and without benomyl.

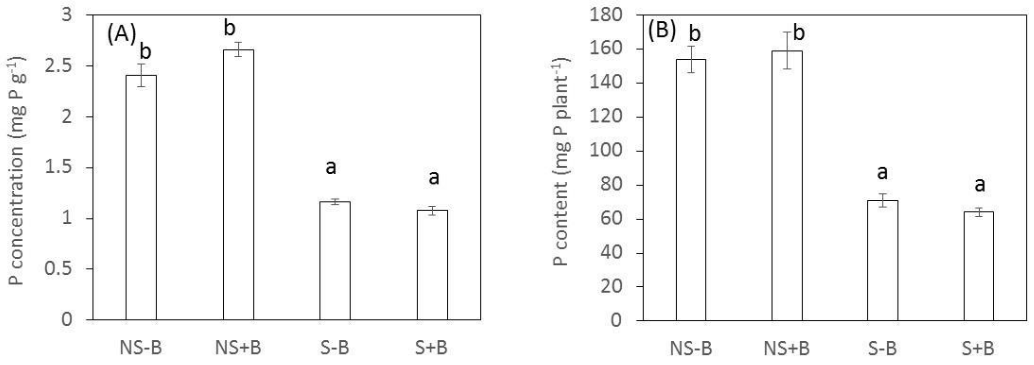

Figure S2 | Effects of N sterilized soil and benomyl on shoot P concentration (A) and shoot P content (B) by maize in Pot 1. Each value is the mean of four replicates (± SE). Different letters denote significant difference ($P < 0.05$) among the treatments. NS-B: non-sterilized soil and no benomyl; NS+B: non-sterilized soil and benomyl; S-B, sterilized soil and no benomyl; S+B, sterilized soil and benomyl.

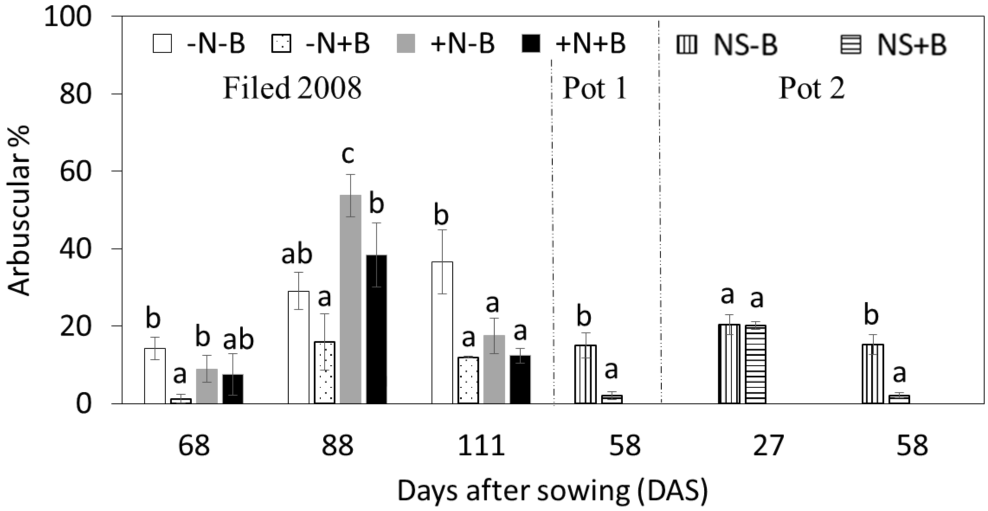

FIGURE S3 | Effects of N fertilizer supply and benomyl on arbuscular (%) by maize at different growth stages in the field experiment in 2008, and in the pot experiment 1 and 2. Each value is the mean of four replicates (± SE). Different letters denote significant difference ($P < 0.05$) among the treatments at each growth stage. -N-B: no N fertilizer and no benomyl; -N+B: no N fertilizer and benomyl; +N-B, N fertilizer and no benomyl; +N+B, N fertilizer and benomyl. DAS: days after sowing for year 2008 (8 = jointing; 88 = silking; 111 = milk: 147 = harvest) NS-B: non-sterilized soil and no-benomyl; NS+B: non-sterilized soil and benomyl in Pot 1 and 2.

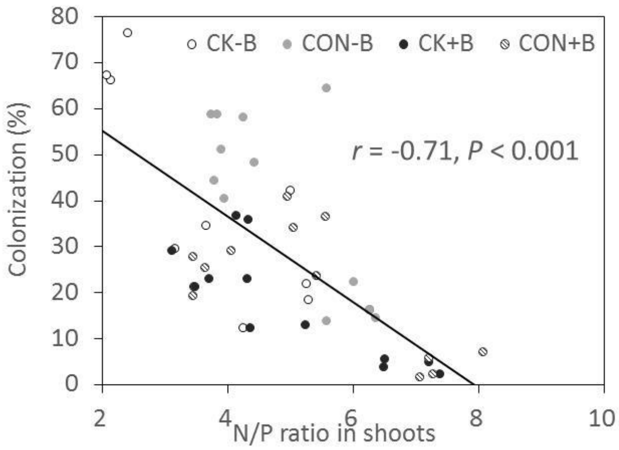

Figure S4 | Relationship between the N/P ratio in shoot of maize and mycorrhizal colonization by native AMF community with and without benomyl at two N levels.
